# Comparative validity and reproducibility of questionnaires in assessing types, frequency, and quantity of sweetened beverage consumption among Mahidol University students

**DOI:** 10.29219/fnr.v70.14204

**Published:** 2026-10-28

**Authors:** Win Lai Khine, Wantanee Kriengsinyos, Tippawan Pongcharoen, Nattapol Tangsuphoom, Aree Prachansuwan, Sabri Bromage

**Affiliations:** 1 Master of Science Program in Nutrition and Dietetics, Institute of Nutrition, Mahidol University, Nakhon Pathom, Thailand; 2 Institute of Nutrition, Mahidol University, Nakhon Pathom, Thailand; 3 Department of Nutrition, Harvard T.H. Chan School of Public Health, Boston, MA, USA

**Keywords:** sugar-sweetened beverages, relative validity, reproducibility, 4-day beverage record, Beverage Intake Questionnaire

## Abstract

**Background:**

Excessive consumption of sugar-sweetened beverages (SSB) is a modifiable risk factor of chronic non-communicable diseases. Accurate and context-specific assessment tools are needed to capture emerging beverage types and to estimate beverage-related sugar intake among university students.

**Objective:**

This study aimed to evaluate the reproducibility and relative validity of a modified Beverage Intake Questionnaire (ModBEVQ) for assessing beverage types, consumption frequency, volume, and total sugar intake of sweetened beverage consumption among Mahidol university students.

**Design:**

A validation study was conducted among 122 students at Mahidol University, Thailand. Reproducibility was assessed by comparing two administrations of the ModBEVQ conducted 1 month apart. Relative validity was assessed by comparing the second BEVQ with a 4-day beverage record (4dBR). Statistical analyses included Spearman’s rank correlation coefficient (*r*) and Intraclass Correlation Coefficients (ICC), and Bland–Altman analysis.

**Results:**

Reproducibility coefficients of the ModBEVQ ranged from 0.27 to 0.84 for beverage volume and from 0.20 to 0.82 for total sugar intake. Relative validity coefficients ranged from 0.16 to 0.65 for beverage volume and from 0.20 to 0.58 for total sugar intake, indicating variable performance across beverage outcome. Bland–Altman analysis demonstrated a small mean bias between the ModBEVQ and the 4dBR. The wide 95% limits of agreement indicated considerable disagreement between the methods at the individual level.

**Conclusion:**

The ModBEVQ demonstrates variable reproducibility and weak-to-moderate relative validity overall, with stronger performance for some beverage outcomes. It may be useful for estimating average beverage volume and sugar intake at the group-level and may have potential applications in epidemiological surveillance and public health monitoring in Thailand, particularly among university students and similar young-adult populations. However, it should not be used to estimate individual beverage intake.

## Popular scientific summary

- Reliable tools are needed to assess beverage and sugar intake among university students.
- This study evaluated the reproducibility and relative validity of a modified Beverage Intake Questionnaire (ModBEVQ) among 122 Mahidol university students.
- The questionnaire performed well for estimating total beverage volume and total sugar intake at the group level, although performance varied by beverage category.
- The ModBEVQ may support research and public-health monitoring among university students, but it should not be used for individual dietary or clinical assessment.

The modern food environment, including the consumption of sugar-sweetened beverages (SSB), is associated with increased prevalence of adverse health outcomes (1, 2). SSB and artificially sweetened beverages (ASBs) significantly contribute to obesity (3, 4) and cardiovascular health issues (5, 6). SSB, such as soft drinks, flavored juices, and energy drinks, contain added sugars, while ASBs, like diet sodas, use low-calorie sweeteners such as aspartame and sucralose. The American Heart Association recommends that men consume no more than 9 teaspoons of added sugar per day and women no more than 6 teaspoons (7). Similarly, the World Health Organization (WHO) advises limiting free sugar intake to less than 10% of total energy, with further benefits observed at levels below 5% or 25 grams (6 teaspoons) per day (8).

Thailand has the highest prevalence of obesity in Asia (9). National reports indicate that Thai people consume approximately 20 teaspoons of sugar per day, exceeding the recommended intake (10) and above the WHO-recommended limit for free sugar consumption (11). Although sugar consumption in Thailand declined in 2020 following sugar taxation policies and the COVID-19 pandemic, beverage consumption patterns continue to evolve as the market expands. Manufacturers have introduced a wider variety of beverages, including sugar-free drinks and products carrying the Healthier Choice Logo, highlighting the need for updated beverage assessment tools (12). University students were selected as the study population because previous research has shown that young adults are among the highest consumers of SSB. Among Association of Southeast Asian Nations (ASEAN) university students, 82% reported daily SSB consumption (13). Studies in Thailand, including one at Khon Kaen University, found that students commonly consumed tea, coffee, and soft drinks 1–3 times per week (14). Another study revealed that Thai dentistry students consumed an average of 42.03 grams of sugar daily from SSB (15). Interestingly, despite knowledge of SSB-associated risks, students at Valaya Alongkorn Rajabhat University continued to consume high-sugar beverages (16). This emphasizes that universities are the main gates to monitor the beverage consumption among young adults.

Traditional methods for assessing intake, such as 24-h recalls and records, are resource-intensive (17, 18). To overcome this challenge, concise beverage frequency questionnaires (BFQ) (19, 20) like BEVQ-15 (21), have been developed to monitor changes in beverage consumption. However, existing BFQs often lack detailed sugar content data or fail to account for emerging beverage categories. The study utilized a modified BEVQ to assess intake of beverages including water. The 4-day Beverage Record (4dBR) provides detailed contemporaneous data against which to assess the relative validity and reliability of the ModBEVQ. In addition, assessing the reproducibility of the tool establishes its consistency for future research and public health initiatives.

This study aims to evaluate the relative validity and reproducibility of the modified BEVQ-15 questionnaire in assessing sweetened beverage types, frequency, and quantity among Mahidol University students.

## Materials and methods

### Study designs

A cross-sectional validation study was conducted from February to April 2024 at Mahidol University’s Salaya campus in Bangkok, Thailand. Participants were recruited during the academic year. Self-administered surveys and records were collected online via bilingual Google Forms (Thai and English).

### Sample size

Validation studies for dietary assessment generally recommend a sample size of at least 100 participants (22–24). To ensure adequate statistical power, the sample size was calculated based on detecting a difference between a predicted correlation of *r*_1_ = 0.6 and a true correlation of *r*_2_ = 0.4. Using Fisher’s Z-transformation, the difference (d) in the standard normal deviates was determined to be 0.269. With a 95% confidence level (*Z*_α_ = 1.96), and 80% power (*Z*_β_ = 0.84), the required sample size (*n*) was calculated (25), $n=\frac{{\left({\text{Z}}_{\alpha }+{\text{Z}}_{\beta }\right)}^{2}}{{\text{d}}^{2}}+3=110$.

This yielded a base sample size of 110 participants. Accounting for a 30% anticipated dropout rate, the required sample size for this research was 158 participants (*N* = *n* / (1-d)).

### Recruitment

Recruitment was conducted via a convenience sampling approach using social media. Eligible Mahidol University students (≥ 18 years old) enrolled in an on-campus program, and excluding those with conditions that could alter habitual intake. A total of 162 participants were initially recruited. Of these, 122 (75.3%) completed all requirements. To assess potential selection bias, baseline characteristics were compared between completers and non-completers, revealing no significant differences.

### Tools

The study utilized a modified version of the BEVQ-15 (21) to better reflect the consumption habits of the Thai student population (26). Modifications (Supplementary Table 1) included expanding beverage categories that are popular among young Thai adults but are not included in the standard BEVQ-15, such as fresh-made smoothies and new alternative drinks (functional beverages). Furthermore, the tool was refined to clearly distinguish between artificially sweetened, unsweetened, and full-sugar beverages. To improve reporting accuracy, the modified questionnaire incorporated visual reference aids of common Thai portion sizes and was provided in a bilingual format.

For data analysis, beverages were categorized into ASBs, SSB, and sugary drinks based on their sugar source and sweetening agents. ASBs included diet or ASBs. SSB included beverages containing added sugars only, such as non-100% fruit juices, regular soft drinks, energy/sports drinks, sweetened teas and coffees, milk-based tea and coffee mixes, and new alternative drinks. Sugary drinks represented a broader category that included all beverages containing free sugars, including both beverages with added sugars and those containing naturally occurring free sugars. Consequently, sugary drinks encompassed SSB together with flavored milks, drinking yogurt, fresh-made smoothies, 100% fruit and vegetable juices, and plant-based milk alternatives, while excluding ASBs, unsweetened beverages, alcoholic beverages, and water.

The 4dBR was a prospective beverage recording tool used to capture beverage intake over a 4-week period. Participants completed one beverage record on one assigned day per week and were instructed to record beverage at the time of consumption or as soon as possible afterward. To represent variations in beverage consumption patterns, participants recorded intake on 3 weekdays and 1 weekend day across the data collection period. The 4dBR was designed to capture all beverages consumed, including water, and participants recorded beverage types, consumed volumes, preparation methods, added ingredients and other relevant details according to the categories provided in Supplementary Table 2. Participants were also instructed to provide real-time photos of beverages consumed to improve the accuracy of reporting.

Pilot testing conducted in December 2023 with 30 participants confirmed the feasibility and clarity of the tools, resulting in minor revisions. The final ModBEVQ included 15 beverage types and was designed as a 20-min online questionnaire, while the 4dBR required 10–15 min per daily entry. The links to the questionnaire and record form were provided to participants every week accordingly with a reminder.

### Data collection

Participants completed a 4dBR once per week over a 4-week period, resulting in four recording days in total (3 weekdays and 1 weekend day), while the ModBEVQ survey was administered twice: during the first (1^st^ ModBEVQ) and final weeks (2^nd^ ModBEVQ). An example of the record and survey timing was attached together in the Participant Information Sheet (PIS). Data collection was conducted online to ensure anonymity and enhance participant convenience.

### Data calculation

For the ModBEVQ, beverage frequency and volume data were analyzed by assigning numerical values to frequency responses, such as 0 for ‘never’, 0.067/day for ‘1–3 times per month’, 0.143/day for ‘1 time per week’, up to 3/day for ‘3 times per day’, and the reported frequency for those consuming more than three times per day. Then, we calculated daily intake (mL/day) by using the formulas:

$$\begin{array}{l} \text{Daily volume intake (dv) mL/day}\;=\;\text{Frequency/day}\;\times \;\text{Amount/frequency},\\ \text{Daily sugar intake (ds) g/day}\;=\;\text{dv (mL/day)}\;\times \;\text{Sugar content per volume (g/mL)}. \end{array}$$

Median sugar content values for individual beverage categories were derived from previous studies on beverage sugar content and the impact of Thailand’s SSB tax (26, 27). To account for variations in beverage formulations following the implementation of Thailand’s SSB tax, selected beverage categories were adjusted using a mean-to-median conversion ratio (1.52:1) based on data from these studies (Supplementary Table 3). The 4dBR data were weighted using a 5:2 ratio of weekdays to weekends to estimate average weekly beverage intake.

### Statistical analysis

Survey data were analyzed using SPSS version 29 (IBM Corp., Armonk, NY, USA). Descriptive statistics included frequencies and percentages for categorical variables, medians with interquartile ranges (IQR) for non-normally distributed numerical data (Shapiro-Wilk *P* < 0.05) and means with standard deviations (SD) for additional representation of central tendency and dispersion. Statistical significance was set at *P* < 0.05.

Spearman’s rank correlation coefficients were used to evaluate the relative validity and reproducibility of the ModBEVQ. The strength of the correlation coefficients was interpreted using thresholds: *r*-values < 0.30 (poor), 0.30–0.49 (moderate), 0.50–0.70 (good), and > 0.70 (strong) (24).

Test-retest reliability was assessed using an Intraclass Correlation Coefficient (ICC) based on a two-way mixed-effects, absolute agreement, single measurement model, with 95% confidence intervals. This model was selected because the purpose was to evaluate the agreement between two administrations of the ModBEVQ and the reliability of a single administration of the ModBEVQ. The average-measure ICC was reported for completeness and comparison with previous validation studies. ICC values were interpreted < 0.50 (poor), 0.50 ≤ ICC < 0.75 (moderate), 0.75 ≤ ICC ≤ 0.90 (good), and > 0.90 (excellent) (28, 29).

Bland–Altman analysis was used to evaluate agreement between the 4dBR and BEVQ2 for volume and sugar intake by calculating the mean bias and 95% limits of agreement (LoA). The acceptability of these limits was determined by comparing the LoA width to a practical threshold, evaluating whether the variation would significantly impact public health intake classifications.

## Results

Relative validity and reproducibility were evaluated for total beverage intake as well as the predefined beverage subgroups (SSB and sugary drinks).

### Study participants

The final analysis (Table 1) included 122 participants aged 18–34 years, of whom 19.7% were male and 80.3% were female. The mean body mass index (BMI) was 22.38 ± 4.86 kg/m^2^. Most participants were undergraduate students (87.7%) and 91.8% were Thai students. Seven participants (5.7%) reported prediabetes or obesity, and six (4.9%) following dietary restrictions. These participants were included in the analysis as their conditions were not considered to significantly interfere with the habitual beverage consumption patterns.

**Table 1:** Demographic and anthropometric characteristics of participants (*n* = 122)

Characteristics	Category/ Range (Minimum–Maximum)	Value
Age (years)^1^	18–34	20.70 ± 2.87
Gender^2^	Male	24 (19.7)
	Female	98 (80.3)
Height (cm)^1^	146–180	162.04 ± 6.76
Weight (kg)^1^	37.1–115.0	59.17 ± 15.49
BMI (kg/m^2^)^1^	15.82–39.33	22.38 ± 4.86
BMI category according to Asia-Pacific classification^2^	Underweight (< 18.5)	29 (23.8)
	Normal (18.5–22.9)	51 (41.8)
	Overweight (23.0–24.9)	13 (10.7)
	Obese I (25.0–29.9)	17 (13.9)
	Obese II (≥ 30.0)	12 (9.8)
Education^2^	Post-graduate	15 (12.3)
	Under-graduate	107 (87.7)
Nationality^2^	Thai students	112 (91.8)
	Non-Thai students	10 (8.2)
Dietary restriction^2^	Yes	6 (4.9)
	No	116 (95.1)
Underlying chronic condition^2^	Yes	7 (5.7)
	No	115 (94.3)

1 Values are expressed as Mean ± Standard Deviation (SD).

2 Values are expressed as frequencies (*n*, %).

### Beverage consumption and sugar intake

Supplementary Table 4 summarizes daily beverage volume (mL/day), and sugar intake (g/day) assessed via the 1^st^ administration of ModBEVQ, 2^nd^ administration of ModBEVQ, and 4dBR. Water had the highest median intake across all methods. Median sugar intake from SSB ranged from 11.68 g/day using the second ModBEVQ to 18.29 g/day using the 4dBR. Total beverage-related sugar intake was generally similar across the three assessments.

### Reproducibility and validity of ModBEVQ

Table 2 presents mean beverage volume and sugar intake estimates, along with Spearman’s correlations for reproducibility, assessed by comparing the first and second ModBEVQ with the 4dBR.

**Table 2:** Mean daily beverage volume and sugar intake and Spearman’s rank correlations for the reproducibility and relative validity of the ModBEVQ

Beverage categories	Daily volume intake (mL/day)					Daily sugar intake (g/day)				
	1^st^ ModBEVQ mean ± SD	2^nd^ ModBEVQ mean ± SD	4dBR mean ± SD	First vs. second ModBEVQ (Reproducibility)	Second ModBEVQ vs. 4dBR (Relative validity)	1^st^ ModBEVQ mean ± SD	2^nd^ ModBEVQ mean ± SD	4dBR mean ± SD	First vs. second ModBEVQ (Reproducibility)	Second ModBEVQ vs. 4dBR (Relative validity)
1. Non-100% fruit juice	37.72 ± 98.25	23.07 ± 62.27	38.03 ± 84.92	0.27**	0.16	2.72 ± 7.07	1.66 ± 4.48	2.66 ± 6.01	0.27**	0.17*
2. Smoothies	57.07 ± 140.93	29.60 ± 67.46	30.21 ± 103.03	0.63**	0.41**	7.79 ± 19.24	4.04 ± 9.21	4.36 ± 14.51	0.63**	0.17
3. 100% fruit juice	11.47 ± 24.49	12.65 ± 51.13	3.77 ± 24.55	0.34**	0.38**	1.09 ± 2.33	1.20 ± 4.86	0.38 ± 2.52	0.34**	0.38**
4. Flavored milk	60.98 ± 97.11	36.11 ± 61.19	38.35 ± 72.70	0.49**	0.36**	5.37 ± 8.55	3.18 ± 5.39	3.19 ± 6.50	0.49**	0.36**
5. Drinking yogurt	27.36 ± 46.70	19.15 ± 41.54	11.13 ± 26.31	0.59**	0.48**	3.20 ± 5.46	2.24 ± 4.86	1.03 ± 2.58	0.59**	0.47**
6. Plant milk	19.47 ± 46.41	12.60 ± 29.98	13.13 ± 35.84	0.52**	0.49**	0.93 ± 2.23	0.60 ± 1.44	0.64 ± 1.87	0.52**	0.49**
7. Soft drink	98.44 ± 169.48	88.54 ± 148.70	76.20 ± 137.34	0.82**	0.62**	8.47 ± 14.58	7.61 ± 12.79	6.49 ± 11.73	0.82**	0.62**
8. Energy/sport drinks	7.15 ± 27.16	5.02 ± 30.57	3.13 ± 16.90	0.39**	0.16	0.69 ± 2.63	0.49 ± 2.97	0.14 ± 1.02	0.39**	0.20*
9. ASB/diet drinks	25.65 ± 46.04	23.84 ± 51.56	23.94 ± 71.07	0.45**	0.39**	–	–	–	–	–
10. Unsweetened drinks	42.59 ± 101.08	24.02 ± 56.26	13.91 ± 39.85	0.39**	0.31**	–	–	–	–	–
11. Sweet tea/coffee	60.18 ± 89.56	51.16 ± 154.52	89.52 ± 148.65	0.60**	0.26**	3.67 ± 5.46	3.12 ± 9.43	5.06 ± 8.76	0.60**	0.25**
12. Milk tea/coffee mix	114.44 ± 162.78	101.25 ± 183.94	116.85 ± 149.17	0.70**	0.41**	6.41 ± 9.12	5.67 ± 10.30	6.54 ± 8.27	0.70**	0.40**
13. New alternative drinks	14.55 ± 33.75	10.31 ± 26.61	17.28 ± 51.99	0.53**	0.24**	0.82 ± 1.89	0.57 ± 1.49	0.75 ± 2.35	0.53**	0.23**
14. Alcoholic drinks	8.96 ± 34.06	6.25 ± 16.83	1.87 ± 20.69	0.54**	0.23*	–	–	–	–	–
15. Water	1304.30 ± 672.40	1367.71 ± 645.73	1342.04 ± 619.33	0.84**	0.65**	–	–	–	–	–
Total (All beverages)	1890.31 ± 746.38	1811.11 ± 733.57	1819.35 ± 554.71	0.74**	0.56**	41.15 ± 37.99	30.39 ± 29.28	31.47 ± 23.59	0.72**	0.61**
ASB	25.65 ± 46.04	23.84 ± 51.56	23.94 ± 71.07	0.45**	0.39**	–	–	–	–	–
SSB (All sugar-sweetened beverages)	332.47 ± 316.62	279.17 ± 338.90	341.01 ± 266.25	0.74**	0.63**	22.77 ± 22.75	19.12 ± 23.07	21.63 ± 17.47	0.76**	0.64**
Sugary drinks	508.81 ± 429.19	389.29 ± 386.31	437.59 ± 282.64	0.70**	0.62**	41.15 ± 37.99	30.39 ± 29.28	31.23 ± 23.46	0.72**	0.62**

** Correlation is significant at the 0.01 level (*P* < 0.01).

* Correlation is significant at the 0.05 level (*P* < 0.05).

Spearman’s correlation (*r*) indicating correlation; *r*-values < 0.30 (poor), 0.30–0.49 (moderate), 0.50–0.70 (good), > 0.70 (strong).

ASB = no. 9, SSB = no. 1+ 7 + 8 + 11 + 12 + 13, Sugary drinks = no. 1+ 2 + 3 + 4 + 5 + 6 + 7 + 8+ 11 + 12 + 13

Artificially sweetened beverages (ASBs) → Sugar-free sodas.

Sugar-sweetened beverages (SSB) → non-100% fruit juices, regular soft drinks, energy/sports drinks, sweetened teas/coffees, milk-based tea/coffee mixes, sugar-added alternative drinks.

Sugary drinks → SSB, flavored milks, smoothies, plant-based milk alternatives. (Excludes ASBs, unsweetened drinks, alcohol, water)

Sugar intake was not calculated for ASB, Unsweetened drinks, Alcoholic drinks, or Water due to zero sugar content or high variability.

Total beverage volume showed strong reproducibility (*r*_s_ = 0.74) and good relative validity (*r*_s_ = 0.56), while total beverage sugar intake also demonstrated strong reproducibility (*r*_s_ = 0.72) and good relative validity (*r*_s_ = 0.61). Among individual beverage categories, water and soft drinks showed the strongest reproducibility correlation for volume intake (*r*_s_ = 0.84 and *r*_s_ = 0.82, respectively). SSB volume and sugar intake exhibited strong reproducibility (*r*_s_ = 0.74 and 0.76, respectively) and good relative validity (*r*_s_ = 0.63 and 0.64, respectively). Soft drinks volume and sugar intake had strong reproducibility (*r*_s_ = 0.82 for both) and good relative validity (*r*_s_ = 0.62 for both). However, fruit juice, energy/sport drinks, and alternative drinks showed weak relative validity correlations.

### Test-retest reliability (ICC)

Table 3 shows ICC values assessing test-retest reliability between 1^st^ ModBEVQ and 2^nd^ ModBEVQ. Both single- and average-measure ICCs were calculated. The single-measure ICCs were used as the primary estimates of reproducibility while average-measure ICCs were presented for comparison.

**Table 3:** Test-retest reliability of the ModBEVQ assessed using showing intraclass correlation coefficients (ICC) for daily volume and sugar intake

Beverage categories	Daily volume intake (mL/day)		Daily sugar intake (g/day)	
	Single measure ICC (95% CI)	Average measure ICC (95% CI)	Single measure ICC (95% CI)	Average measure ICC (95% CI)
Non-100% fruit juice	0.06 (−0.12 to 0.23)	0.11 (−0.27 to 0.38)	0.06 (−0.12 to 0.23)	0.11 (−0.27 to 0.38)
Smoothies	0.66 (0.54 to 0.75)	0.79 (0.70 to 0.86)	0.66 (0.54 to 0.75)	0.79 (0.70 to 0.86)
100% fruit juice	0.07 (−0.11 to 0.24)	0.13 (−0.25 to 0.39)	0.07 (−0.11 to 0.24)	0.13 (−0.25 to 0.39)
Flavored milk	0.44 (0.29 to 0.57)	0.61 (0.44 to 0.73)	0.44 (0.28 to 0.57)	0.61 (0.44 to 0.73)
Drinking yogurt	0.48 (0.33 to 0.61)	0.65 (0.50 to 0.76)	0.48 (0.33 to 0.61)	0.65 (0.50 to 0.76)
Plant milk	0.62 (0.50 to 0.72)	0.77 (0.66 to 0.84)	0.62 (0.50 to 0.72)	0.77 (0.66 to 0.84)
Soft drink	0.72 (0.60 to 0.78)	0.83 (0.75 to 0.88)	0.71 (0.60 to 0.78)	0.83 (0.75 to 0.88)
Energy/sport drinks	0.04 (−0.14 to 0.22)	0.08 (−0.31 to 0.36)	0.04 (−0.14 to 0.22)	0.08 (−0.31 to 0.36)
ASB/diet drinks	0.32 (0.15 to 0.47)	0.48 (0.26 to 0.64)	–	–
Unsweetened drinks	0.31 (0.14 to 0.46)	0.47 (0.25 to 0.63)	–	–
Sweet tea/coffee	0.29 (0.12 to 0.45)	0.45 (0.21 to 0.62)	0.29 (0.12 to 0.45)	0.45 (0.21 to 0.62)
Milk tea/coffee mix	0.74 (0.65 to 0.81)	0.85 (0.79 to 0.90)	0.74 (0.65 to 0.81)	0.85 (0.79 to 0.90)
New alternative drinks	0.50 (0.35 to 0.62)	0.67 (0.52 to 0.77)	0.50 (0.35 to 0.62)	0.66 (0.52 to 0.77)
Alcoholic drinks	0.29 (0.12 to 0.45)	0.45 (0.22 to 0.62)	–	–
Water	0.85 (0.79 to 0.89)	0.92 (0.88 to 0.94)	–	–
Total (All beverages)	0.74 (0.65 to 0.81)	0.85 (0.79 to 0.90)	0.61 (0.48 to 0.71)	0.76 (0.65 to 0.83)
ASB	0.32 (0.15 to 0.47)	0.26 to 0.64	–	–
SSB (All sugar-sweetened beverages)	0.58 (0.45 to 0.69)	0.73 (0.62 to 0.81)	0.58 (0.45 to 0.69)	0.74 (0.62 to 0.81)
Sugary drinks	0.59 (0.46 to 0.70)	0.74 (0.63 to 0.82)	0.61 (0.48 to 0.71)	0.76 (0.65 to 0.83)

ICCs were estimated using a two-way mixed-effects, absolute-agreement, single-measurement and are reported with 95% confidence intervals.

Intraclass correlation coefficients (ICC) indicating reliability; ICC < 0.50, poor, 0.50 ≤ ICC < 0.75, moderate; 0.75 ≤ ICC ≤ 0.90, good; and ICC > 0.90, excellent.

For beverage volume intake, water demonstrated the highest reliability (ICC = 0.85), followed by milk tea/coffee mixes (ICC = 0.74). Total beverage volume (ICC = 0.74), SSB volume (ICC = 0.58), and sugary drinks volume (ICC = 0.59) showed moderate to good reliability. In contrast, fruit juices (ICC = 0.07) and energy/sports drinks (ICC = 0.04) indicated poor reliability.

For sugar intake, milk tea/coffee mixes and soft drinks had good reliability (ICC = 0.74 and 0.71, respectively). Total beverage sugar intake also showed moderate reliability (ICC = 0.61), while flavored milk, drinking yogurt, and plant milk had poor reliability. Energy/sports drinks and fruit juices had low ICCs, reflecting poor test-retest consistency.

### Bland–Altman analysis

Supplementary Table 5 details the mean differences and LoA, calculated as Upper/Lower Limit = Mean ± 1.96 SD, reflecting the agreement between the ModBEVQ and the 4dBR.

The Bland–Altman plots (Fig. 1) demonstrated a relatively small negative mean difference (bias) between the second administration of the ModBEVQ and the 4dBR. The mean bias for total beverage volume was −8.25 mL/day, with 95% LoA ranging from −1151.56 to 1135.06 mL/day. The corresponding mean biases were −1.09 g/day (95% LoA: −47.91 to 45.73 g/day) for total beverage sugar intake, −61.84 mL/day (95% LoA: −658.13 to 534.45 mL/day) for SSB volume, −2.51 g/day (95% LoA: −39.51 to 34.49 g/day) for SSB sugar intake, −48.31 mL/day (95% LoA: −702.93 to 606.31 mL/day) for sugary-drinks volume, and −0.84 g/day (95% LoA: −47.18 to 45.49 g/day) for sugary-drinks sugar intake. Overall, the ModBEVQ slightly underestimated beverage volume and sugar intake compared with the 4dBR. However, the 95% LoA were relatively wide, particularly for beverage volume estimates, indicating greater variability between the two methods at the individual level. The variability of the difference appeared to increase with increasing intake, suggesting poorer agreement between the two methods among participants with higher beverage consumption. A small proportion of observations fell outside the 95% LoA (4.1 to 7.4% across outcomes), with detailed confidence intervals for the mean bias, LoA, and numbers of observations outside the LoA presented in Supplementary Table 6.

**Fig. 1:**
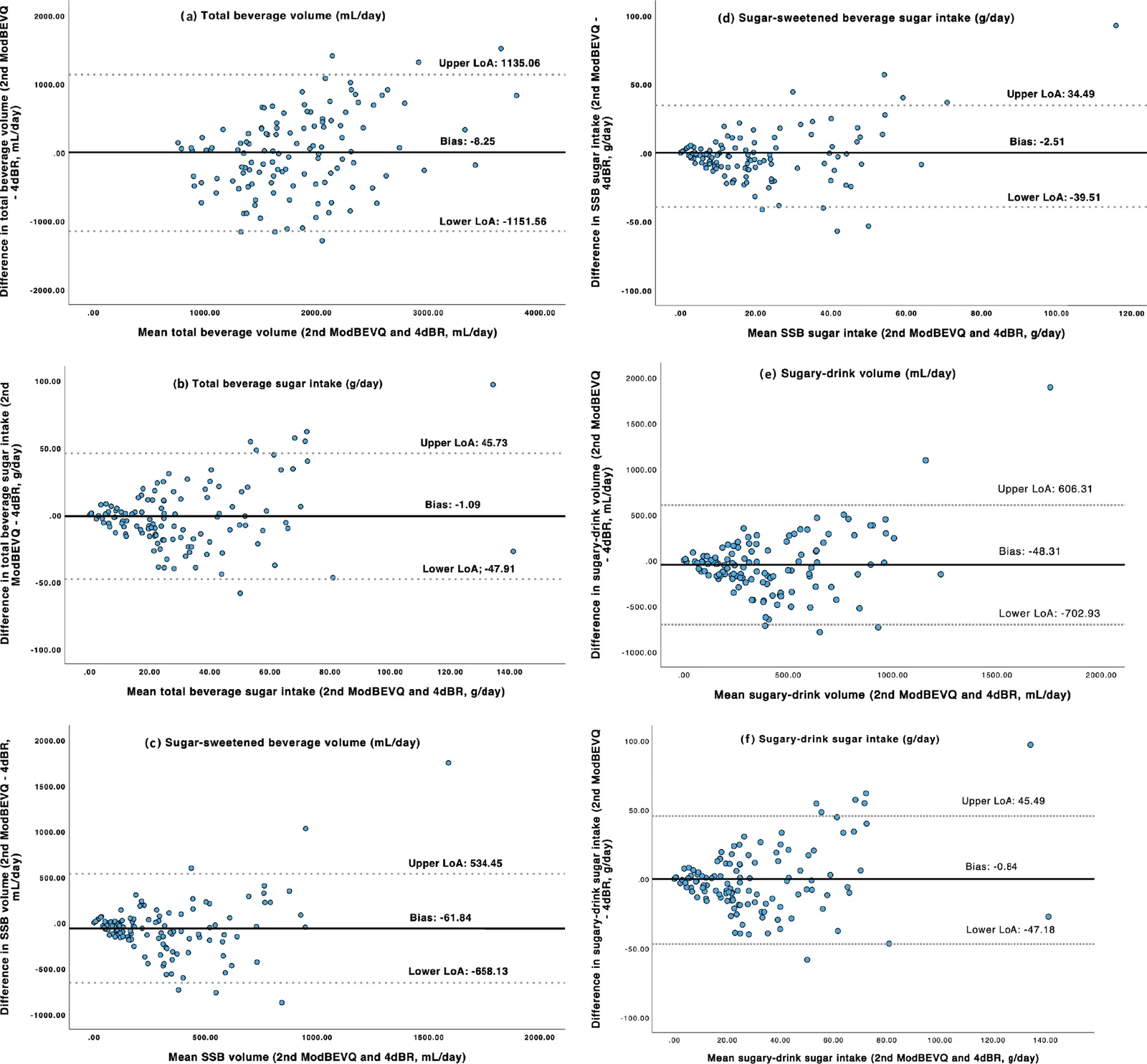
Bland–Altman Plots comparing the second administration of the modified Beverage Intake Questionnaire (2nd ModBEVQ) with the 4-Day Beverage Records (4dBR). Panels show (a) total beverage volume, (b) total beverage sugar intake, (c) sugar-sweetened beverage volume, (d) sugar-sweetened beverage sugar intake, (e) sugary-drink volume, (f) sugary-drink sugar intake. Each point represents one participant (*n* = 122). The x-axis represents the mean of the two methods, and the y-axis represents the difference between two methods (2^nd^ ModBEVQ – 4dBR). The solid line indicates the mean difference (bias), and the dashed lines indicate the 95% limits of agreement (mean difference ± 1.96 SD).

## Discussion

This study evaluated the reproducibility and relative validity of the ModBEVQ among university students in Thailand. The questionnaire demonstrated strong reproducibility, but moderate relative validity for total beverage volume and total beverage-related sugar intake, although its performance varied across individual beverage categories. Frequently consumed beverages, particularly water, soft drinks, and milk tea/coffee mixes, generally showed stronger reproducibility, test-retest reliability, and relative validity than beverages consumed less regularly, consistent with previous BEVQ validation studies (19–21).

The poorer performance observed for non-100% fruit juice, energy/sport drinks, and some emerging beverage categories may partly reflect their infrequent or episodic consumption. Intake of these beverages may vary considerably between assessment periods, making usual consumption more difficult to estimate using either a questionnaire or a short-term beverage record. Such variability may contribute to the lower reproducibility and relative validity observed for these beverage categories.

The Bland–Altman analysis showed small mean biases for most outcomes, suggesting limited systematic differences between the ModBEVQ and the 4dBR at the group level. However, the ModBEVQ systematically underestimated SSB volume, as the 95% confidence interval for the mean bias did not include zero. One possible explanation is misclassification of beverage when completing the ModBEVQ, particularly when participants had difficulty distinguishing sweetened from unsweetened variants or assigning beverages to the appropriate SSB category. Differences in recall period, portion-size estimation, and day-to-day variation in SSB consumption may also have contributed to this discrepancy. Because these potential sources of error were not directly evaluated, these explanations should be interpreted cautiously. In addition, the relatively wide LoA across outcomes indicated considerable disagreement between the methods at the individual level. Therefore, although the ModBEVQ may provide reasonable estimates of average beverage volume and sugar intake for groups, it lacks sufficient precision for individual dietary or clinical assessment. The dispersion of the differences appeared to increase at higher intake levels, suggesting that agreement may be poorer among participants with greater beverage consumption. Because proportional bias was not formally tested, this pattern should be interpreted cautiously. Some disagreement may also reflect limitations of the 4dBR, including day-to-day variation in beverage consumption and the possibility that the recording period did not fully present habitual intake, particularly for beverage categories consumed irregularly.

The Mod BEVQ was adapted to reflect beverage types, serving sizes, and consumption patterns relevant to Thai university students. Administration through an online survey platform may facilitate data collection and reduce administrative burden in large studies. This approach is consistent with previous research suggesting that dietary assessment instruments adapted to the foods, beverages, language, and serving sizes of a specific population may provide more relevant intake estimates than unmodified instruments (19).

Unlike some previous beverage questionnaires that focused mainly on beverage volume or energy intake, this study assessed both beverage volume and total beverage-related sugar intake. Total sugar includes both sugars naturally present in certain beverages and added sugars; therefore, it provides an estimate of overall sugar exposure from beverages but cannot distinguish between naturally occurring and added sugars.

This study used a prospective 4dBR, with participants completing one recording day per week over four consecutive weeks (3 weekdays and 1 weekend day). This design was intended to capture both weekday–weekend variation and some week-to-week variation in beverage intake. In contrast, the FG-FFQ study among Chinese middle-school students used four non-consecutive 24-h dietary records collected over 3 months as its reference method (30). The FG-FFQ study estimated added sugars from SSB and applied calibration equations to improve correspondence with the dietary records, whereas this study estimated total beverage sugar intake and used conventional reproducibility and agreement analyses. Future research could examine whether calibration equations improve the estimates obtained from the ModBEVQ.

Similarly, the Canadian validation study (18) using a 7-day food record also reported better performance for frequently consumed beverages than for beverages consumed sporadically. Differences between studies may reflect variations in beverage culture, questionnaire design, reference methods, recording periods, and participant characteristics.

This study has several strengths. The ModBEVQ was adapted to locally relevant beverage categories and assessed both beverage volume and beverage-related total sugar intake. Reproducibility, test-retest reliability and relative validity were evaluated using complementary statistical approaches, including Spearman’s rank correlations, ICCs, and Bland–Altman analysis. Furthermore, the prospective 4dBR included both weekday and weekend intake, and category-specific analyses identified beverage groups requiring further refinement.

Several limitations should also be acknowledged. Firstly, although the final sample exceeded the minimum required sample size, the 24.7% attrition rate may have introduced selection bias. Because the characteristics of participants who completed and did not complete the study were not compared, the effect of attrition could not be determined. Secondly, the sample was predominantly female and was recruited from one university, limiting generalizability to male students, students in other institutions, and other young adult populations. Thirdly, data collection coincided with Thailand’s hot season, and university midterm examination period. These factors may have influenced beverage consumption pattern and therefore may limit the generalizability of the findings to other seasons and academic periods. Fourthly, both the ModBEVQ and the 4dBR relied on self-reported and were therefore susceptible to recall bias, portion-size estimation errors, and changes in consumption behavior during recording intake. The 4dBR is not an error-free reference method and may not adequately represent habitual intake of episodically consumed beverages, including fruit juices, energy drinks, and alcoholic beverages. Special occasions, social events, and other atypical consumption days were also not excluded from the records. Finally, the wide Bland–Altman LoA indicate that individual beverage intake cannot be estimated with sufficient precision using the ModBEVQ.

The findings of this study have several implications for both epidemiological research and public-health practice. The ModBEVQ may be useful for estimating average beverage volume and beverage-related sugar intake at the group level among university students and similar young-adult populations. It may therefore have potential applications in epidemiological surveillance, campus-based health-promotion programs, and evaluation of interventions intended to reduce SSB consumption.

However, the questionnaire should not be used for precise individual dietary assessment or clinical decision-making. The poorer performance observed for infrequently consumed beverages identifies specific categories requiring further refinement. Improvements could include clearer beverage examples, updated portion-size images, more detailed prompts for emerging beverages, and shorter recall periods for episodically consumed products.

Before broader implementation in Thailand, the ModBEVQ should be validated in larger and more diverse populations, including participants from different institutions, geographic regions, age groups, ethnic backgrounds, and seasons. Future studies should also consider additional recording days, real-time image-assisted recording, and statistical calibration methods to improve estimates for beverages consumed infrequently or irregularly.

## Conclusion

The ModBEVQ demonstrated good relative validity and strong reproducibility for estimating total beverage volume and total beverage-related sugar intake, although performance varied across individual beverage categories. Small mean biases support its potential use for estimating average intake at the group level, whereas the wide LoA indicate insufficient precision for individual dietary or clinical assessment. The ModBEVQ may have potential applications in epidemiological surveillance and public-health monitoring in Thailand, particularly among university students and similar young-adult populations. Further refinement is needed for infrequently consumed beverages, such as fruit juices and energy drinks, and additional validation should be conducted in more diverse populations and across different seasons.

## Supplementary Material


