# Supplementary material for: Comparative validity and reproducibility of questionnaires in assessing types, frequency, and quantity of sweetened beverage consumption among Mahidol University students
Source: Food Nutr Res. 2026 Oct 28;70:10.29219/fnr.v70.14204. doi: 10.29219/fnr.v70.14204 (PMC13732515; doi:10.29219/fnr.v70.14204)
Supplement: Supplementary file 1 [file FNR-70-14204-s1.docx]

**Supplementary Table 1.** Comparison of the original BEVQ-15* and the ModBEVQ

| **Feature** | **Original BEVQ-15*** | **Modified BEVQ** |
| --- | --- | --- |
| Local specialty drinks | Juice/milk categories | Included freshly prepared smoothie beverage containing sweetened milk or yogurt |
| Functional/new Drinks | Not categorized | Included an additional category for functional and alternative beverages |
| Beverage Specificity | Broad beverage categories | Sweetened dairy beverages categorized separately to reflect beverage types available in Thailand |
| Sugar/ Sweetener Status | General reporting | Beverages categorized as diet/ artificially Sweetened (zero-sugar), unsweetened (plain), or full-sugar |
| Portion Estimation | Standard US serving sizes | Visual reference images of common Thai serving sizes included |
| Language | English | Available in both Thai and English |

### * The original BEVQ-15 was developed by (21) Hedrick et al. The ModBEVQ was adapted to reflect beverage consumption patterns and commonly available beverage products among Thai university students.

### **Supplementary Table 2.** Schedule of the 4-day beverage record and the first and second ModBEVQ administrations.

| Weeks | Beverage record (4dBR) | ModBEVQ |
| --- | --- | --- |
| Week 1 (W-1) | One recording day (weekday) | 1^st^ ModBEVQ |
| Week 2 (W-2) | One recording day (weekend) |  |
| Week 3 (W-3) | One recording day (weekday) |  |
| Week 4 (W-4) | One recording day (weekday) | 2^nd^ ModBEVQ |

### Participants completed one beverage record per week over four consecutive weeks, comprising three weekday records and one weekend record. The specific recording day varied according to each participant’s schedule.

### **Supplementary Table 3.** Sugar content estimation and calculations of individual beverage categories used in the ModBEVQ.

|  | **Beverage category** | **Typical serving size** | **Mean**  **sugar content (g/serve)** | **Mean**  **sugar content (g/100mL)** | **Mean: median ratio^1^** | **Sugar value used in ModBEVQ (g/100mL)** |
| --- | --- | --- | --- | --- | --- | --- |
| 1 | Non-100% fruit juice (50-99%) | 230 mL/can | 24.33 | 10.58 | 1.16:1 | 9.10 |
|  | Non-100% fruit juice (25-49%) | 180 mL/ bottle | 19.04 | 10.58 | 1.58:1 | 6.70 |
|  | Non-100% fruit juice (<25%) | 200 mL/box | 21.16 | 10.58 | 1.82:1 | 5.80 |
|  | Average non-100% fruit juice |  |  |  | 1.52:1 | 7.20* |
| 2 | Fresh made smoothie juice | 650 mL/glass | 135.0 | 20.77 | 1.52:1 | 13.65* |
| 3 | 100%fruit juice |  |  |  |  | 9.50* |
| 4 | Flavored milk | 200 mL/box | 39.18 | 19.59 |  | 8.80* |
| 5 | Yogurt/ sour milk | 180 mL/box | 18.23 | 10.12 | 0.86:1 | 11.70* |
| 6 | Plant-based milk |  |  |  |  | 4.80* |
| 7 | Carbonated drink | 325 mL/can | 40.88 | 12.58 | 1.46:1 | 8.60* |
| 8 | Non-carbonated drink (energy drink) | 100 mL/bottle | 27.11 | 27.11 | 2.79:1 | 9.70* |
| 9 | Diet drink |  |  |  |  | 0.00 |
| 10 | Unsweetened drinks |  |  |  |  | 0.00 |
| 11 | Sweet tea and coffee (Fresh iced tea/coffee) | 650 mL/glass | 48.04 | 7.39 | 1.23:1 | 6.10* |
| 12 | Milk tea and coffee (ready to drink coffee) | 180 mL/can | 12.61 | 7.01 | 1.25:1 | 5.60* |
| 13 | New Alternative drinks | 500 mL/glass | 91.10 | 18.22 |  | 5.60* |
|  | Functional/ beauty drinks |  |  |  |  | 4.50 |
| 14 | Alcoholic |  |  |  |  | Not estimated |
| 15 | Water |  |  |  |  | 0.00 |

Sugar content values and adjustment factors were derived from published studies evaluating beverage sugar content and changes following implementation of Thailand’s sugar-sweetened beverage tax (26,27).

^1^ The mean-to-median ratio was calculated as mean sugar content divided by median sugar content and was applied to selected beverage categories.

* Median sugar content values were used in the ModBEVQ calculations.

### **Table 2.** Mean **daily beverage volume and sugar intake and Spearman’s rank correlations for the reproducibility and relative validity of the ModBEVQ.**

| Beverage Categories | Daily volume intake (mL/day) | | | | | Daily sugar intake (g/day) | | | | |
| --- | --- | --- | --- | --- | --- | --- | --- | --- | --- | --- |
|  | 1^st^ ModBEVQ  mean ± SD | 2^nd^ ModBEVQ  mean ± SD | 4dBR  mean  ± SD | First vs. second ModBEVQ  (Reproducibility) | Second ModBEVQ vs 4dBR  (Relative validity) | 1^st^ ModBEVQ  mean ± SD | 2^nd^ ModBEVQ  mean ± SD | 4dBR  mean  ± SD | First vs. second ModBEVQ  (Reproducibility) | Second ModBEVQ vs 4dBR  (Relative validity) |
| 1. Non-100% fruit juice | 37.72  ± 98.25 | 23.07  ± 62.27 | 38.03  ± 84.92 | 0.27** | 0.16 | 2.72  ± 7.07 | 1.66  ± 4.48 | 2.66  ± 6.01 | 0.27** | 0.17* |
| 2. Smoothies | 57.07  ±140.93 | 29.60  ± 67.46 | 30.21  ± 103.03 | 0.63** | 0.41** | 7.79  ± 19.24 | 4.04  ± 9.21 | 4.36  ± 14.51 | 0.63** | 0.17 |
| 3. 100% fruit juice | 11.47  ± 24.49 | 12.65  ± 51.13 | 3.77  ± 24.55 | 0.34** | 0.38** | 1.09  ± 2.33 | 1.20  ± 4.86 | 0.38  ± 2.52 | 0.34** | 0.38** |
| 4. Flavoured milk | 60.98  ± 97.11 | 36.11  ± 61.19 | 38.35  ± 72.70 | 0.49** | 0.36** | 5.37  ± 8.55 | 3.18  ± 5.39 | 3.19  ± 6.50 | 0.49** | 0.36** |
| 5. Drinking yogurt | 27.36  ± 46.70 | 19.15  ± 41.54 | 11.13  ± 26.31 | 0.59** | 0.48** | 3.20  ± 5.46 | 2.24  ± 4.86 | 1.03  ± 2.58 | 0.59** | 0.47** |
| 6. Plant milk | 19.47 ± 46.41 | 12.60 ± 29.98 | 13.13 ± 35.84 | 0.52** | 0.49** | 0.93  ± 2.23 | 0.60  ± 1.44 | 0.64  ± 1.87 | 0.52** | 0.49** |
| 7. Soft drink | 98.44  ± 169.48 | 88.54  ± 148.70 | 76.20  ± 137.34 | 0.82** | 0.62** | 8.47  ± 14.58 | 7.61  ± 12.79 | 6.49  ± 11.73 | 0.82** | 0.62** |
| 8. Energy/ Sport drinks | 7.15  ± 27.16 | 5.02  ± 30.57 | 3.13  ± 16.90 | 0.39** | 0.16 | 0.69  ± 2.63 | 0.49  ± 2.97 | 0.14  ± 1.02 | 0.39** | 0.20* |
| 9. ASB/ Diet drinks | 25.65  ± 46.04 | 23.84  ± 51.56 | 23.94  ± 71.07 | 0.45** | 0.39** | - | - | - | - | - |
| 10. Unsweetened drinks | 42.59  ± 101.08 | 24.02  ± 56.26 | 13.91  ± 39.85 | 0.39** | 0.31** | - | - | - | - | - |
| 11. Sweet tea/ coffee | 60.18  ± 89.56 | 51.16  ± 154.52 | 89.52  ± 148.65 | 0.60** | 0.26** | 3.67  ± 5.46 | 3.12  ± 9.43 | 5.06  ± 8.76 | 0.60** | 0.25** |
| 12. Milk tea/ coffee mix | 114.44  ± 162.78 | 101.25  ± 183.94 | 116.85  ± 149.17 | 0.70** | 0.41** | 6.41  ± 9.12 | 5.67  ± 10.30 | 6.54  ± 8.27 | 0.70** | 0.40** |
| 13. New alternative drinks | 14.55  ± 33.75 | 10.31  ± 26.61 | 17.28  ± 51.99 | 0.53** | 0.24** | 0.82  ± 1.89 | 0.57  ± 1.49 | 0.75  ± 2.35 | 0.53** | 0.23** |
| 14. Alcoholic drinks | 8.96  ± 34.06 | 6.25  ± 16.83 | 1.87  ± 20.69 | 0.54** | 0.23* | - | - | - | - | - |
| 15. Water | 1304.30  ± 672.40 | 1367.71  ± 645.73 | 1342.04  ± 619.33 | 0.84** | 0.65** | - | - | - | - | - |
| Total (All beverages) | 1890.31  ± 746.38 | 1811.11  ± 733.57 | 1819.35  ± 554.71 | 0.74** | 0.56** | 41.15  ± 37.99 | 30.39  ± 29.28 | 31.47  ± 23.59 | 0.72** | 0.61** |
| ASB | 25.65  ± 46.04 | 23.84  ± 51.56 | 23.94  ± 71.07 | 0.45** | 0.39** | - | - | - | - | - |
| SSB (All sugar-sweetened beverages) | 332.47  ± 316.62 | 279.17  ± 338.90 | 341.01  ± 266.25 | 0.74** | 0.63** | 22.77  ± 22.75 | 19.12  ± 23.07 | 21.63  ± 17.47 | 0.76** | 0.64** |
| Sugary drinks | 508.81  ± 429.19 | 389.29  ± 386.31 | 437.59  ± 282.64 | 0.70** | 0.62** | 41.15  ± 37.99 | 30.39  ± 29.28 | 31.23  ± 23.46 | 0.72** | 0.62** |

** Correlation is significant at the 0.01 level (p-value < 0.01). * Correlation is significant at the 0.05 level (p-value < 0.05).

Spearman’s correlation (r) indicating correlation; r values < 0.30 (poor), 0.30–0.49 (moderate), 0.50–0.70 (good), > 0.70 (strong)

**ASB** = no. 9**, SSB** = no. 1+ 7 + 8 + 11 + 12 + 13, **Sugary drinks** = no. 1+ 2 + 3 + 4 + 5 + 6 + 7 + 8+ 11 + 12 + 13

Artificially sweetened beverages (ASBs) 🡪 Sugar-free sodas.

Sugar-sweetened beverages (SSB) 🡪 non-100% fruit juices, regular soft drinks, energy/sports drinks, sweetened teas/coffees, milk-based tea/coffee mixes, sugar-added alternative drinks.

Sugary drinks 🡪 SSB, flavored milks, smoothies, plant-based milk alternatives. (Excludes ASBs, unsweetened drinks, alcohol, water.)

Sugar intake was not calculated for ASB, Unsweetened drinks, Alcoholic drinks, or Water due to zero sugar content or high variability.

### **Table 3.** Test-retest reliability of the ModBEVQ assessed using showing intraclass correlation coefficients (ICC) for **daily volume and sugar intake.**

| **Beverage categories** | **Daily volume intake (mL/day)** | | **Daily sugar intake (g/day)** | |
| --- | --- | --- | --- | --- |
|  | Single measure  ICC (95% CI) | Average measure  ICC (95% CI) | Single measure  ICC (95% CI) | Average measure  ICC (95% CI) |
| **Non-100% fruit juice** | 0.06 (-0.12 to 0.23) | 0.11 (-0.27 to 0.38) | 0.06 (-0.12 to 0.23) | 0.11 (-0.27 to 0.38) |
| **Smoothies** | 0.66 (0.54 to 0.75) | 0.79 (0.70 to 0.86) | 0.66 (0.54 to 0.75) | 0.79 (0.70 to 0.86) |
| **100% fruit juice** | 0.07 (-0.11 to 0.24) | 0.13 (-0.25 to 0.39) | 0.07 (-0.11 to 0.24) | 0.13 (-0.25 to 0.39) |
| **Flavoured milk** | 0.44 (0.29 to 0.57) | 0.61 (0.44 to 0.73) | 0.44 (0.28 to 0.57) | 0.61 (0.44 to 0.73) |
| **Drinking yogurt** | 0.48 (0.33 to 0.61) | 0.65 (0.50 to 0.76) | 0.48 (0.33 to 0.61) | 0.65 (0.50 to 0.76) |
| **Plant milk** | 0.62 (0.50 to 0.72) | 0.77 (0.66 to 0.84) | 0.62 (0.50 to 0.72) | 0.77 (0.66 to 0.84) |
| **Soft drink** | 0.72 (0.60 to 0.78) | 0.83 (0.75 to 0.88) | 0.71 (0.60 to 0.78) | 0.83 (0.75 to 0.88) |
| **Energy/ Sport drinks** | 0.04 (-0.14 to 0.22) | 0.08 (-0.31 to 0.36) | 0.04 (-0.14 to 0.22) | 0.08 (-0.31 to 0.36) |
| **ASB/ Diet drinks** | 0.32 (0.15 to 0.47) | 0.48 (0.26 to 0.64) | - | - |
| **Unsweetened drinks** | 0.31 (0.14 to 0.46) | 0.47 (0.25 to 0.63) | - | - |
| **Sweet tea/ coffee** | 0.29 (0.12 to 0.45) | 0.45 (0.21 to 0.62) | 0.29 (0.12 to 0.45) | 0.45 (0.21 to 0.62) |
| **Milk tea/ coffee mix** | 0.74 (0.65 to 0.81) | 0.85 (0.79 to 0.90) | 0.74 (0.65 to 0.81) | 0.85 (0.79 to 0.90) |
| **New alternative drinks** | 0.50 (0.35 to 0.62) | 0.67 (0.52 to 0.77) | 0.50 (0.35 to 0.62) | 0.66 (0.52 to 0.77) |
| **Alcoholic drinks** | 0.29 (0.12 to 0.45) | 0.45 (0.22 to 0.62) | - | - |
| **Water** | 0.85 (0.79 to 0.89) | 0.92 (0.88 to 0.94) | - | - |
| **Total (All beverages)** | 0.74 (0.65 to 0.81) | 0.85 (0.79 to 0.90) | 0.61 (0.48 to 0.71) | 0.76 (0.65 to 0.83) |
| **ASB** | 0.32 (0.15 to 0.47) | 0.26 to 0.64 | - | - |
| **SSB (All sugar-sweetened beverages)** | 0.58 (0.45 to 0.69) | 0.73 (0.62 to 0.81) | 0.58 (0.45 to 0.69) | 0.74 (0.62 to 0.81) |
| **Sugary drinks** | 0.59 (0.46 to 0.70) | 0.74 (0.63 to 0.82) | 0.61 (0.48 to 0.71) | 0.76 (0.65 to 0.83) |

ICCs were estimated using a two-way mixed-effects, absolute-agreement, single-measurement and are reported with 95% confidence intervals.

Intraclass correlation coefficients (ICC) indicating reliability; ICC < 0.50, poor, 0.50 ≤ ICC < 0.75, moderate; 0.75 ≤ ICC ≤ 0.90, good; and ICC > 0.90, excellent.

### **Supplementary Table 4. Median and interquartile range (IQR) of beverage daily volume intake (mL/day) and sugar intake (g/day) across 1^st^ ModBEVQ, 2^nd^ ModBEVQ, and 4dBR**

| Beverage Categories | Daily volume intake (mL/day) | | | | | | Daily sugar intake (g/day) | | | | | |
| --- | --- | --- | --- | --- | --- | --- | --- | --- | --- | --- | --- | --- |
|  | 1^st^ ModBEVQ | | 2^nd^ ModBEVQ | | 4dBR | | 1^st^ ModBEVQ | | 2^nd^ ModBEVQ | | 4dBR | |
|  | Median | IQR  (Q1-Q3) | Median | IQR  (Q1-Q3) | Median | IQR  (Q1-Q3) | Median | IQR  (Q1-Q3) | Median | IQR  (Q1-Q3) | Median | IQR  (Q1-Q3) |
| Non-100% fruit juice | 13.40 | 36.03  (0.00-36.03) | 0.00 | 20.10  (0.00-20.10) | 0.00 | 30.36  (0.00-30.36) | 0.96 | 2.59  (0.00-2.59) | 0.00 | 1.45  (0.00-1.45) | 0.00 | 2.38  (0.00-2.38) |
| Smoothies | 24.12 | 43.55  (0.00-43.55) | 0.00 | 33.71  (0.00-33.71) | 0.00 | 0.00  (0.00-0.00) | 3.29 | 5.94  (0.00-5.94) | 0.00 | 4.60  (0.00-4.60) | 0.00 | 0.00  (0.00-0.00) |
| 100% fruit juice | 0.00 | 13.40  (0.00-13.40) | 0.00 | 0.00  (0.00-0.00) | 0.00 | 0.00  (0.00-0.00) | 0.00 | 1.27  (0.00-1.27) | 0.00 | 0.00  (0.00-0.00) | 0.00 | 0.00  (0.00-0.00) |
| Flavoured milk | 16.75 | 73.63  (0.00-73.63) | 15.08 | 36.03  (0.00-36.03) | 0.00 | 50.00  (0.00-50.00) | 1.47 | 6.48  (0.00-6.48) | 1.33 | 3.17  (0.00-3.17) | 0.00 | 2.92  (0.00-2.92) |
| Drinking yogurt | 9.38 | 37.71  (0.00-37.71) | 5.70 | 20.10  (0.00-20.10) | 0.00 | 0.00  (0.00-0.00) | 1.10 | 4.18  (0.00-4.18) | 0.67 | 2.35  (0.00-2.35) | 0.00 | 0.00  (0.00-0.00) |
| Plant milk | 0.00 | 16.75  (0.00-16.75) | 0.00 | 16.75  (0.00-16.75) | 0.00 | 0.00  (0.00-0.00) | 0.00 | 0.80  (0.00-0.80) | 0.00 | 0.80  (0.00-0.80) | 0.00 | 0.00  (0.00-0.00) |
| Soft drink | 33.50 | 176.99  (1.51-178.50) | 32.83 | 83.00  (0.00-83.00) | 0.00 | 114.29  (0.00-114.29) | 2.88 | 15.22  (0.13-15.35) | 2.83 | 7.14  (0.00-7.14) | 0.00 | 9.38  (0.00-9.38) |
| Energy/ Sport drinks | 0.00 | 0.00  (0.00-0.00) | 0.00 | 0.00  (0.00-0.00) | 0.00 | 0.00  (0.00-0.00) | 0.00 | 0.00  (0.00-0.00) | 0.00 | 0.00  (0.00-0.00) | 0.00 | 0.00  (0.00-0.00) |
| ASB/ Diet drinks | 0.00 | 30.15  (0.00-30.15) | 0.00 | 23.45  (0.00-23.45) | 0.00 | 0.00  (0.00-0.00) | - | - | - | - | - | - |
| Unsweetened drinks | 0.00 | 36.80  (0.00-36.83) | 0.00 | 21.53  (0.00-21.53) | 0.00 | 0.00  (0.00-0.00) | - | - | - | - | - | - |
| Sweet tea/ coffee | 18.43 | 89.25  (0.00-89.25) | 23.45 | 33.50  (0.00-33.50) | 0.00 | 146.13  (0.00-146.13) | 1.13 | 5.44  (0.00-5.44) | 1.43 | 2.04  (0.00-2.04) | 0.00 | 7.60  (0.00-7.60) |
| Milk tea/ coffee mix | 46.48 | 171.36  (0.00-171.36) | 32.16 | 149.94  (0.00-149.94) | 74.43 | 185.71  (0.00-185.71) | 2.60 | 9.60  (0.00-9.60) | 1.80 | 8.40  (0.00-8.40) | 4.00 | 10.17  (0.00-10.17) |
| New alternative drinks | 0.00 | 12.73  (0.00-12.73) | 0.00 | 2.51  (0.00-2.51) | 0.00 | 0.00  (0.00-0.00) | 0.00 | 0.72  (0.00-0.72) | 0.00 | 0.14  (0.00-0.14) | 0.00 | 0.00  (0.00-0.00) |
| Alcoholic drinks | 0.00 | 0.84  (0.00-0.84) | 0.00 | 0.00  (0.00-0.00) | 0.00 | 0.00  (0.00-0.00) | - | - | - | - | - | - |
| Water | 1200.00 | 1000.00  (800.00-1800.00) | 1250.00 | 900.00  (900.00-1800.00) | 1347.62 | 860.12  (832.74-1692.86) | - | - | - | - | - | - |
| Total (All beverages) | 1851.81 | 939.46  (1366.43-2305.89) | 1700.88 | 940.07  (1299.98-2240.05) | 1788.10 | 739.77  (1429.29-2169.05) | 27.37 | 40.06  (16.48-56.54) | 21.76 | 33.36  (10.51-43.86) | 27.76 | 30.65  (13.69-44.35) |
| ASB | 0.00 | 30.15  (0.00-30.15) | 0.00 | 23.45  (0.00-23.45) | 0.00 | 0.00  (0.00-0.00) | - | - | - | - | - | - |
| SSB (All sugar-sweetened beverages) | 245.38 | 373.18  (82.08-455.26) | 180.49 | 321.66  (60.92-382.58) | 267.03 | 430.95  (114.29-545.24) | 15.77 | 27.32  (5.22-32.54) | 11.68 | 21.81  (3.88-25.68) | 18.29 | 21.85  (7.92-29.78) |
| Sugary drinks | 370.93 | 516.81  (213.26-730.07) | 268.10 | 415.26  (130.80-546.06) | 369.67 | 440.42  (201.79-642.20) | 27.37 | 40.06  (16.48-56.54) | 21.78 | 33.36  (10.51-43.86) | 27.22 | 30.43  (13.69-44.13) |

Values Represented by Median (P25, P75).

Artificially sweetened beverages (ASBs) **🡪** Sugar-free sodas.

Sugar-sweetened beverages (SSB) 🡪 non-100% fruit juices, regular soft drinks, energy/sports drinks, sweetened teas/coffees, milk-based tea/coffee mixes, sugar-added alternative drinks.

Sugary drinks 🡪 SSB, flavored milks, smoothies, plant-based milk alternatives. (Excludes ASBs, unsweetened drinks, alcohol, water.)

Sugar intake was not calculated for ASB, Unsweetened drinks, Alcoholic drinks, or Water due to zero sugar content or high variability.

### **Supplement Table 5.** Mean **daily beverage volume intake (mL/day) and sugar intake (g/day), with mean difference** ± SD and percentage difference between methods.

| Beverage Categories | Daily volume intake (mL/day) | | | | | Daily sugar intake (g/day) | | | | |
| --- | --- | --- | --- | --- | --- | --- | --- | --- | --- | --- |
|  | 1^st^ ModBEVQ  mean ± SD | 2^nd^ ModBEVQ  mean ± SD | 4dBR  mean  ± SD | Difference  2^nd^ ModBEVQ-1^st^ ModBEVQ  mean difference ± SD  (% difference) | Difference  2^nd^ ModBEVQ-4dBR  mean difference  ± SD  (% difference) | BEVQ1  mean  ± SD | BEVQ2  mean  ± SD | 4dBR  mean  ± SD | Difference  2^nd^ ModBEVQ-1^st^ ModBEVQ  mean difference  ± SD  (% difference) | Difference  2^nd^ ModBEVQ-4dBR  mean difference ± SD  (% difference) |
| Non-100% fruit juice | 37.72  ± 98.25 | 23.07  ± 62.27 | 38.03  ± 84.92 | -14.66 ± 112.83 (-38.9%) | -14.97 ± 100.85  (-39.4%) | 2.72  ± 7.07 | 1.66 ± 4.48 | 2.66  ± 6.01 | -1.06 ± 8.12  (-39.0%) | -1.00 ± 7.19  (-37.6%) |
| Smoothies | 57.07  ± 140.93 | 29.60  ± 67.46 | 30.21  ± 103.03 | -27.47 ± 91.49  (-48.1%) | -0.61 ± 63.30  (-2.0%) | 7.79  ± 19.24 | 4.04  ± 9.21 | 4.36  ± 14.51 | -3.75 ± 12.49  (-48.1%) | -0.32 ± 8.98  (-7.3%) |
| 100% fruit juice | 11.47  ± 24.49 | 12.65  ± 51.13 | 3.77  ± 24.55 | 1.18 ± 54.75  (10.3%) | 8.88 ± 34.73  (235.5%) | 1.09  ± 2.33 | 1.20  ± 4.86 | 0.38  ± 2.52 | 0.11 ± 5.20  (10.1%) | 0.82 ± 3.24  (215.8%) |
| Flavoured milk | 60.98  ± 97.11 | 36.11  ± 61.19 | 38.35  ± 72.70 | -24.86 ± 85.97  (-40.8%) | -2.23 ± 71.99  (-5.8%) | 5.37  ± 8.55 | 3.18  ± 5.39 | 3.19  ± 6.50 | -2.19 ± 7.57  (-40.8%) | -0.02 ± 6.33  (-0.6%) |
| Drinking yogurt | 27.36  ± 46.70 | 19.15  ± 41.54 | 11.13  ± 26.31 | -8.21 ± 44.95  (-30.0%) | 8.02 ± 40.85  (72.1%) | 3.20  ± 5.46 | 2.24  ± 4.86 | 1.03  ± 2.58 | -0.96 ± 5.26  (-30.0%) | 1.21 ± 4.93  (117.5%) |
| Plant milk | 19.47  ± 46.41 | 12.60  ± 29.98 | 13.13  ± 35.84 | -6.87 ± 34.11  (-35.3%) | -0.53 ± 31.69  (-4.0%) | 0.93  ± 2.23 | 0.60  ± 1.44 | 0.64  ± 1.87 | -0.33 ± 1.64  (-35.5%) | -0.04 ± 1.83  (-6.3%) |
| Soft drink | 98.44  ± 169.48 | 88.54  ± 148.70 | 76.20  ± 137.34 | -9.89 ± 122.40  (-10.0%) | 12.35 ± 121.72  (16.2%) | 8.47  14.58 | 7.61  12.79 | 6.49  11.73 | -0.85 ± 10.53  (-10.0%) | 1.13 ± 10.25  (17.4%) |
| Energy/ Sport drinks | 7.15  ± 27.16 | 5.02  ± 30.57 | 3.13  ± 16.90 | -2.13 ± 39.99  (-29.8%) | 1.89 ± 35.25  (60.4%) | 0.69  ± 2.63 | 0.49  ± 2.97 | 0.14  ± 1.02 | -0.21 ± 3.88  (-30.4%) | 0.34 ± 3.14  (242.9%) |
| ASB/ Diet drinks | 25.65  ± 46.04 | 23.84  ± 51.56 | 23.94  ± 71.07 | -1.81 ± 57.08  (-7.1%) | -0.10 ± 64.59  (-0.4%) | - | - | - | - | - |
| Unsweetened drinks | 42.59  ± 101.08 | 24.02  ± 56.26 | 13.91  ± 39.85 | -18.57 ± 96.11  (-43.6%) | 10.11 ± 56.03  (72.7%) | - | - | - | - | - |
| Sweet tea/ coffee | 60.18  ± 89.56 | 51.16  ± 154.52 | 89.52  ± 148.65 | -9.02 ± 150.44  (-15.0%) | -38.36 ± 196.13  (-42.9%) | 3.67  ± 5.46 | 3.12  ± 9.43 | 5.06  ± 8.76 | -0.55 ± 9.18  (-15.0%) | -1.93 ± 11.84  (-38.2%) |
| Milk tea/ coffee mix | 114.44  ± 162.78 | 101.25  ± 183.94 | 116.85  ± 149.17 | -13.18 ± 125.50  (-11.5%) | -15.59 ± 191.96  (-13.3%) | 6.41  ± 9.12 | 5.67  ± 10.30 | 6.54  ± 8.27 | -0.74 ± 7.03  (-11.5%) | -0.87 ± 10.75  (-13.3%) |
| New alternative drinks | 14.55  ± 33.75 | 10.31  ± 26.61 | 17.28  51.99 | -4.43 ± 30.46  (-30.4%) | -7.16 ± 56.20  (-41.4%) | 0.82  ± 1.89 | 0.57  ± 1.49 | 0.75  ± 2.35 | -0.25 ± 1.71  (-30.5%) | -0.18 ± 2.74  (-24.0%) |
| Alcoholic drinks | 8.96  ± 34.06 | 6.25  ± 16.83 | 1.87  ± 20.69 | -2.71 ± 31.95  (-30.2%) | 4.38 ± 18.31  (234.2%) | - | - | - | - | - |
| Water | 1304.30  ± 672.40 | 1367.71  ± 645.73 | 1342.04  ± 619.33 | 63.40 ± 363.50  (4.9%) | 25.67 ± 523.05  (1.9%) | - | - | - | - | - |
| Total (All beverages) | 1890.31  ± 746.38 | 1811.11  ± 733.57 | 1819.35  ± 554.71 | -79.21± 533.18  (-4.2%) | -8.25 ± 583.32  (-0.5%) | 41.15  ± 37.99 | 30.39  ± 29.28 | 31.47  ± 23.59 | -10.76 ± 30.03  (-26.1%) | -1.09 ± 23.89  (-3.5%) |
| ASB | 25.65  ± 46.04 | 23.84  ± 51.56 | 23.94  ± 71.07 | -1.81 ± 57.08  (-7.1%) | -0.10 ± 64.59  (-0.4%) | - | - | - | - | - |
| SSB (All sugar-sweetened beverages) | 332.47  ± 316.62 | 279.17  ± 338.90 | 341.01  ± 266.25 | -53.30 ± 298.77  (-16.0%) | -61.84 ± 304.23  (-18.1%) | 22.77  ± 22.75 | 19.12  ± 23.07 | 21.63  ± 17.47 | -3.65 ± 20.98  (-16.0%) | -2.51± 18.88  (-11.6%) |
| All sugary drinks | 508.81  ± 429.19 | 389.29  ± 386.31 | 437.59  ± 282.64 | -119.53  ± 371.35  (-23.5%) | -48.31  ± 333.99  (-11.0%) | 41.15  ± 37.99 | 30.39  ± 29.28 | 31.23  ± 23.46 | -10.76  ± 30.03  (-34.5%) | -0.84  ± 23.64  (-2.7%) |

Values are presented as mean ± SD. Differences are presented as the mean paired difference ± SD of the differences, with the percentage difference in parentheses. For reproducibility, percentage difference was calculated as (second ModBEVQ−first ModBEVQ)/first ModBEVQ×100. For relative validity, it was calculated as (second ModBEVQ−4dBR)/4dBR×100.

### **Supplement Table 6.** Bland-Altman agreement analysis between the second administration of the Modified Beverage Intake Questionnaire (2^nd^ ModBEVQ) and the 4-day beverage record (4dBR), including mean bias, 95% confidence intervals (CI) for the bias and limits of agreement (LoA), and observations outside the LoA (n = 122).

| **Outcome** | **Mean bias**  **(95% CI)** | **Lower LoA**  **(95% CI)** | **Upper LoA**  **(95% CI)** | **Below lower LoA**  **n (%)** | **Above upper LoA**  **n (%)** | **Total outside LoA**  **n (%)** |
| --- | --- | --- | --- | --- | --- | --- |
| **Total beverage volume (mL/day)** | - 8.25  (-111.75 to 92.26) | -1151.56  (-1330.80 to -972.32) | 1135.06  (955.82 to 1314.30) | 3 (2.5%) | 3 (2.5%) | 6 (5.0%) |
| **Total beverage sugar intake (g/day)** | -1.09  (-5.32 to 3.15) | -47.91  (-55.25 to -40.57) | 45.73  (38.39 to 53.07) | 1 (0.8%) | 6 (4.9%) | 7 (5.7%) |
| **SSB volume (mL/day)** | -61.84  (-115.86 to -7.82) | -658.13  (-751.67 to -564.59) | 534.45  (440.91 to 627.99) | 3 (2.5%) | 3 (2.5%) | 6 (5.0%) |
| **SSB sugar intake**  **(g/day)** | -2.51  (-5.86 to 0.84) | -39.47  (-45.29 to -33.65) | 34.45  (28.63 to 40.27) | 4 (3.3%) | 5 (4.1%) | 9 (7.4%) |
| **Sugary-drink volume (mL/day)** | -48.31  (-107.58 to 10.97) | -702.93  (-805.70 to -600.16) | 606.31  (503.54 to 709.08) | 3 (2.5%) | 2 (1.6%) | 5 (4.1%) |
| **Sugary-drink sugar intake (g/day)** | -0.84  (-5.04 to 3.35) | -47.17  (-54.44 to -39.90) | 45.32  (38.05 to 52.59) | 1 (0.8%) | 6 (4.9%) | 7 (5.7%) |

Abbreviations: LoA, limits of agreement; CI, confidence interval; SSB, sugar-sweetened beverage. Mean bias was calculated as the difference between the 2^nd^ ModBEVQ and 4dBR. Lower and upper LoA were calculated as mean bias ± 1.96 SD. Percentages represent the proportion of participants whose difference fell outside the 95% limits of agreement.
